# Effectiveness of a Two-Tier Family-Oriented Intervention in Enhancing the Family Functioning and Care Capacity of the Family Caregivers of Stroke Survivors: Protocol for a Randomized Controlled Trial

**DOI:** 10.2196/16703

**Published:** 2021-05-28

**Authors:** Vivian Weiqun Lou, Jennifer Yee Man Tang, Gary Kui Kai Lau, Terry Yat Sang Lum, Kenneth Fong, Rachel Wai Tung Ko, Clio Yuen Man Cheng, Joyce Yinqi Fu, Eddie Siu Lun Chow, Angus Chun Kwok Chu, Elsie Hui, Winnie Wing Ling Ng, Felix Hon Wai Chan, C C Luk, T K Kwok

**Affiliations:** 1 Sau Po Centre on Ageing The University of Hong Kong Hong Kong Hong Kong; 2 Department of Social Work and Social Administration The University of Hong Kong Hong Kong Hong Kong; 3 Department of Medicine The University of Hong Kong Hong Kong Hong Kong; 4 The State Key Laboratory of Brain and Cognitive Sciences The University of Hong Kong Hong Kong Hong Kong; 5 Department of Rehabilitation Sciences The Hong Kong Polytechnic University Hong Kong Hong Kong; 6 Department of Medicine and Geriatrics Tuen Mun Hospital Hospital Authority Hong Kong Hong Kong; 7 Department of Medicine and Geriatrics Shatin Hospital Hospital Authority Hong Kong Hong Kong; 8 Department of Medicine and Geriatrics Fung Yiu King Hospital Hospital Authority Hong Kong Hong Kong; 9 Queen Mary Hospital Hospital Authority Hong Kong Hong Kong; 10 Division of Rehabilitation Medicine Tung Wah Hospital Hospital Authority Hong Kong Hong Kong

**Keywords:** two-tier family-oriented intervention, family functioning, family caregivers, stroke survivors, randomized controlled trial

## Abstract

**Background:**

Stroke has profound impacts on families. Often, family members, including stroke survivors and the person who takes up the role of the primary caregiver, would encounter demands on finances, rehabilitation arrangement, and even conflicts. Hence, a family-oriented intervention is expected to enable families to rebuild internal and external resources to achieve optimal rehabilitation and community reintegration.

**Objective:**

This study aims to describe a design of a two-tier family-oriented care management intervention for enhancing the family functioning and care capacity of the caregivers of stroke survivors.

**Methods:**

The two-tier care management intervention was guided by a standardized protocol conducted by trained professional care managers (first tier) with the support of trained volunteers (second tier), which lasted for 8-12 weeks. Participants were recruited through collaborating hospitals according to inclusion and exclusion criteria. In order to examine the effectiveness and cost-effectiveness of the two-tier care management intervention, a two-arm randomization multicenter study was designed, including an active comparison group, which was guided by a standardized protocol conducted by trained volunteers. Dyadic participants, including both stroke survivors and their primary caregivers for both groups, were invited to participate in a questionnaire survey using standardized and purposefully developed measures 3 times: before the intervention, immediately after the intervention, and 2 months after the intervention. The primary outcome was family functioning measured by the Family Role Performance Scale and Family Assessment Device-General Functioning Scale. The secondary outcomes included caregiving burden, depressive symptoms, care management strategies, and the incremental cost-effectiveness ratio.

**Results:**

Recruitment began in January 2017 and was completed at the end of April 2019. Data collection was completed at the end of March 2020. As of March 2020, enrollment has been completed (n=264 stroke caregivers). A total of 200 participants completed the baseline questionnaires. We aim to publish the results by mid-2021.

**Conclusions:**

This study successfully developed a two-tier care management protocol that aims to enhance the family functioning of the caregivers of stroke survivors. Guided by a standardized protocol, this family-oriented two-tier intervention protocol was found to be feasible among Chinese families.

**Trial Registration:**

ClinicalTrials.gov NCT03034330; https://ichgcp.net/clinical-trials-registry/NCT03034330

**International Registered Report Identifier (IRRID):**

RR1-10.2196/16703

## Introduction

### Background and Rationale

Stroke has profound impacts on families. In particular, after stroke survivors return home from discharge, they often rely on family members to meet their daily needs for care and support [1,2]. Often, family members, including stroke survivors and the person who takes up the role of the primary caregiver, would be under high stress to face the demands of supporting the activity of daily living, the instrumental activity of daily living, financial and emotional support, rehabilitation arrangement, etc. During this sudden and unexpected caregiving journey, stroke caregivers need to not only equip themselves with knowledge and skills to provide hands-on care on a daily basis but also adjust their roles and functions within the family context. The process of rehabilitation after stroke is often long and challenging, and feelings of frustration, depression, and even family conflicts are common. A study showed that stroke survivor characteristics and family conflicts surrounding recovery was associated with mental distress and physical ill health [3]. In turn, increased psychological distress and poor family function can undermine the stroke survivor’s recovery and rehabilitation process [1,4,5]. Apart from the direct impact of stroke caregivers, poor recovery and rehabilitation of stroke survivors impose a significant financial burden on the health care system [6].

Stroke accounts for 30%-50% of admissions to long-term residential care homes in Hong Kong [6]. Hence, interventions targeting family dynamics are vital. Interventions focusing on primary caregivers revealed the importance of enhancing self-efficacy in reducing the burden and enhancing their well-being at the individual level [7-11]. However, as we argued above, stroke affects the entire family and not only the primary caregivers. Hence, we developed a family-oriented intervention to support families with stroke survivors. A family is the most important social unit that is expected to provide care and support when family members feel sick or ill. When a person experiences a stroke, the family as a whole is affected, and therefore, interventions that enable family functioning as a whole are required.

Research has shown that a volunteer-led community education program successfully improved stroke knowledge [12], indicating that volunteers are untapped resources that can be integrated into interventions. However, studies have shown that a professional-led intervention group produced better outcomes in preventing mood disorders among stroke patients as compared to the volunteer support group [13]. Considering that professional-led and volunteer-led interventions could have unique roles, our research team designed a two-tier care management intervention protocol. In the first tier, trained professionals focused on conducting the assessment, conducting family-oriented interventions such as work-family balance, family communication, and family conference, and assigning and supervising volunteer-led intervention sessions. In the second tier, trained volunteers focused on interventions on enhancing stroke knowledge, enabling exercise, etc. By integrating the two-tier care management intervention, professionals and volunteers could supplement each other to optimize the intervention intensity and to maximize cost-effectiveness.

In summary, our research team developed a two-tier family-oriented intervention that aims to enhance the family functioning of the caregivers of stroke survivors and it has 3 unique features. These features are (1) a family-oriented intervention that focuses on family functioning, (2) a two-tier care management approach consisting of trained professionals (first tier) and trained volunteers (second tier) purposefully designed, and (3) last but not the least, a family-oriented care management intervention, starting from needs assessment, followed by the care plan, implementation, and review.

### Objectives and Hypothesis

The main objective of this 8-12-week randomized controlled trial is to investigate the effectiveness and cost-effectiveness of a two-tier family-oriented intervention in enhancing the family functioning of caregivers of stroke survivors. We hypothesized that a two-tier family-oriented intervention for caregivers of stroke survivors can enhance their family functioning and care capacity as compared to a volunteer-led control intervention.

## Methods

### Study Setting

This is a two-armed, multicenter, double-blind randomized controlled trial (NCT03034330). Stroke survivors and their primary caregivers (up to n=300) were included and randomized in a 1:1 allocation ratio to a two-tier family-oriented care manager–led intervention group (up to n=150) and a volunteer-led control group (up to n=150). In this study, the research team collaborated with 5 local hospitals in the Hong Kong West Cluster, New Territories East Cluster, and New Territories West Cluster and 3 nongovernmental organizations (NGOs) in the Southern, Shatin, and Tuen Mun districts with varied settings. Three on-site teams established the infrastructure to provide direct rehabilitation and support services to stroke patients. The details of the partnering NGOs are listed in Table 1.

**Table 1:** Details of the study sites.

District	Setting	Services
Southern	District Elderly Community Centre	Providing support services to healthy, vulnerable, and frail older adults living in the community and family caregivers
Shatin	Home Support Team of the Integrated Discharge Support Program for Elderly Patients	Providing postdischarge support services for older adult patients, such as meal delivery, household cleaning, home assessment, and modification
Tuen Mun	Community Rehabilitation Day Centre	Providing both professional and psychosocial rehabilitation services to discharged patients with stroke, neurological, or physical impairments

### Ethical Approval

We obtained ethical approval from the Institutional Review Board of The University of Hong Kong/Hospital Authority Hong Kong West Cluster (UW 16-1019), The Joint Chinese University of Hong Kong-New Territories East Cluster Clinical Research Ethics Committee (2016.679-T), and New Territories West Cluster Research Ethics Committee (NTWC/CREC/16123). The clinical trial was registered at the United States National Institutes of Health (clinicaltrials.gov; NCT03034330). Informed written consent was obtained from all participants included in the study. This study started in June 2016 and ended in March 2020.

### Inclusion Criteria

All caregivers of stroke survivors identified by the NGOs or referred by the hospitals were recruited if they (1) were Cantonese-speaking adults aged 18 years or older, (2) had a family member who had a stroke (ischemic or hemorrhagic stroke) at the age of 18 years or older, (3) provided care or were with a stroke survivor for no less than 10 hours per week (including time supervising a domestic helper) after discharge from the acute hospital, and (4) reported significant caregiver burden (a score ≥6 using the four-item Zarit Caregiver Burden Interviews [14]), depressive mood (a score ≥2 using the Patient Health Questionnaire-2 [15]), or family dysfunction (a score ≥6 using 4 items selected from the Family Caregiver Conflict Scale [FCCS] for Stroke [16]). These 3 parameters were chosen because this study aims to enhance the family functioning and care capacity of stroke caregivers. The inclusion criteria for stroke survivors were (1) being a Cantonese-speaking adult aged 18 years or older, (2) having a family caregiver participating in this study, (3) being able to communicate with interventionists and interviewers, and (4) being competent to provide written informed consent.

### Exclusion Criteria

Caregivers were excluded if they were diagnosed with Alzheimer disease or other types of dementia (clinically diagnosed), or were suffering from acute health conditions (eg, conditions caused by a virus, infection, injury, misuse of drugs or medication) that prevented them from providing caregiving support. As this intervention involves strong engagement and commitment from caregivers, people with cognitive impairments are not suitable for this study. Acute health conditions include cancers and other major illnesses such as stroke and a broken bone that negatively affect the caregivers’ physical and mental conditions, which prevented them from providing caregiving or joining the intervention. Stroke survivors (1) who had a transient ischemic attack without a major ischemic or hemorrhagic stroke, (2) whose family caregiver refused to participate in this study, (3) who were not able to communicate with interventionists and interviewers, or (4) who were not competent to give written informed consent (eg, illiterate or with cognitive impairment) were excluded from this study.

### Sample Size Calculation

We used the G*Power 3 software (Psychonomic Society, Inc) to determine the minimum sample size required for obtaining a significant medium effect size of family functioning, given α=.05 and statistical power of 0.80, with a two-sided significance of .05. While we estimated the sample size, we could only take references from similar relevant studies reported in the literature that were available. References were taken from studies using individual-based outcomes such as the readmission rate of stroke survivors [17], depressive symptoms of caregivers [18], family role performance [19], and caregiver burden [4]. We admit that this is a limitation of our study. Based on a previous study, readmission rates within 6 months after discharge were significantly lower among patients who received follow-up home visits (26%-34%) compared to those who received standard aftercare only (44%) [17]. Besides, stroke accounts for 30%-50% of admissions to long-term residential care homes in Hong Kong [6]. To the best of our knowledge, since no intervention has utilized both care managers and volunteers in providing support to stroke families, we hypothesize that our designed intervention will lower the readmission rates of stroke survivors. As per this, we hypothesized that 15% of the stroke survivors whose caregivers were in the intervention group will be readmitted to a hospital or admitted to a residential care facility within 6 months after discharge, whereas 30% of the stroke survivors whose caregivers were in the control group will be readmitted to a hospital or admitted to a residential care facility over the same period. The resulting sample size estimate was 140 dyads per group; however, to ensure a sufficient effect size, and considering attrition, a sample size of 150 dyads per group will be used. We estimated that the attrition rate will be 25% based on the risk of second stroke occurrence within 1 year [20] as well as the fact that the previous intervention study lasted for around 6 months [17].

### Screening, Randomization, and Intervention Allocation

Stroke survivors and their primary caregivers were first to be identified by physicians in the abovementioned 5 hospitals. Potential dyads were referred to the 3 NGOs using a standardized referral form developed by the research team. Stroke survivors and their primary caregivers were also identified from the pool of existing service users of the 3 NGOs. The care team consisted of trained social workers from the 3 NGOs who performed screening using a standardized screening form that comprised data on demographics, family functioning, caregiving burden, depressive symptoms, and willingness to join. Participants were recruited if they met the inclusion criteria, passed at least one threshold in the areas of family functioning, caregiving burden, or depressive symptoms, and showed a willingness to join.

After recruitment, trained social workers from the 3 NGOs notified the research team to conduct randomization. To avoid bias and to ensure an equal number in each group within each study site, group allocation was determined using computer-generated block randomization with a block size of 4 by using SPSS Statistics (version 23.0, IBM Corp). A member of the research team informed the care managers about the subject code for group allocation. The participants and researchers were blinded to the group assignment. The care managers and volunteers were not blinded to the group assignment, because they needed to know the participants’ group assignment to provide interventions.

Upon randomization, trained care managers were assigned to different cases by the NGOs’ internal control while 2-3 trained volunteers were assigned to the same group depending on their availability and gender. The research team was cautious about the gender of the volunteers; female caregivers may find it difficult to face many male volunteers due to traditional values, while male caregivers may be more willing to receive support from male volunteers.

Three strategies were adopted to ensure that the intervention and control groups were separated throughout the study. First, both interventions were guided by a standardized protocol conducted by the trained interventionists independently. Second, interventions were home-based, which prevented participants from meeting with each other during the intervention period. Last but not the least, the research team served as a safe guide during the monthly field visit to secure protocol compliance.

### Protocol Development

The research team developed a standardized protocol consisting of 3 guidelines, that is, a Care Manager Manual (with 4 chapters), an Intervention Group Protocol for Volunteers (with 13 chapters), and a Control Group Protocol for Volunteers (with 6 chapters) to facilitate the intervention program. The research team provided training to the care managers and volunteers separately. First, we conducted a 4-day (32-hour) training session for 9 care managers (3 from each NGO) in 2016, covering the topics of the medical aspects of stroke, the intervention and assessment framework, care management, family therapy, coping with stress, problem-solving, stroke rehabilitation, and family intervention skills. Second, we provided a 5-day (40-hour) training workshop to 46 volunteers recruited by the 3 NGOs, focusing on the impact of stroke, psychoeducation, in-home exercise training, personal care techniques, counseling skills, swallowing and communications, intervention assessment, and code of practice. The number of volunteers recruited was estimated by care managers of the 3 NGOs based on the intervention protocol and resource management.

### Intervention Group

Both groups followed a standardized protocol designed by the research team. The intervention group received a two-tier family-oriented care manager–led intervention consisting of a trained social worker (care manager) and trained volunteers for 2-3 months.

### Control Group

**Figure 1:**
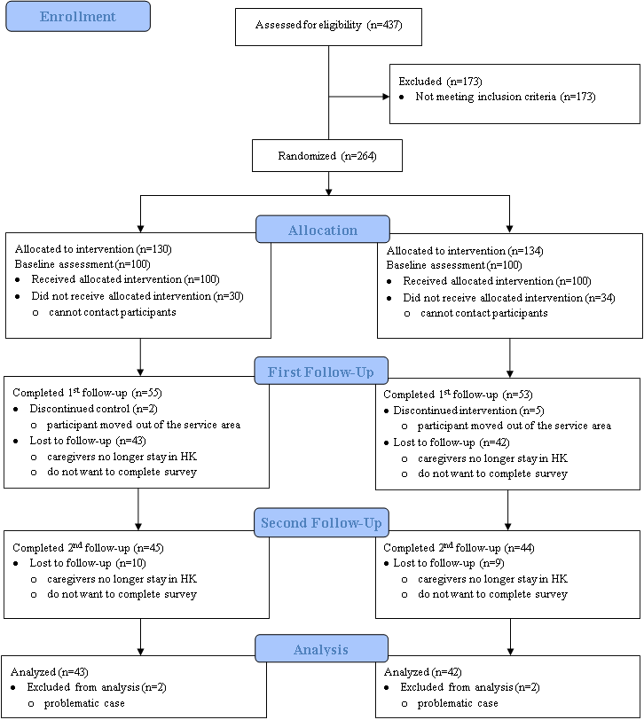
CONSORT flow diagram of the randomized controlled trial with 2 groups. HK: Hong Kong.

The control group received a volunteer-led psychosocial education for 2-3 months. Care managers did not provide any direct intervention for the participants in the control group. Stroke survivors were not involved in the control group intervention. All participants completed a questionnaire at 3 different time points (Figure 1). A baseline assessment (T0) was conducted by care managers or volunteers before the start of the intervention. The first follow-up (T1) was conducted by a trained research assistant immediately after completion of the intervention, and the second follow-up (T2) was conducted by a trained research assistant at 2 months after completion of the intervention. Time points were chosen to better evaluate the effectiveness of the intervention and to reduce the attrition rate.

### Intervention

Stroke caregiver participants in the intervention group received the following interventions.

#### Two-Tier Family-Oriented Care Manager–Led Intervention

The intervention was individualized and tailor-made according to the caregivers’ needs assessment results. Care managers conducted an initial family needs assessment with caregivers to determine the care plan. Baseline assessments and a family genogram were used to assess caregivers’ needs. Measures covered demographic information, stroke knowledge, care management strategies, family conflicts, family functioning, social network, caregiver burden, physical and mental health, and depressive mood. The intervention lasted for 2-3 months with 6-10 weekly sessions at the homes of the caregivers or stroke survivors. Each session lasted for 60-90 minutes. The flexibility in the duration was provided based on the consideration that some participating families were more willing to share and discuss in detail, as evidenced in the pilot experiences. The research team visited each study site every 4 weeks to ensure project progress and held a full team meeting with the 3 NGOs every 12 weeks to share good practices.

The care managers determined the intensity of the intervention after the initial family needs assessment. The weekly sessions selected out of 22 choices are listed in Table 2. The trained care manager provided the first tier of the intervention, consisting of (1) the family needs assessment (session 1), (2) family support and counseling (sessions 2, 3, 4, 5, 6, and 7), (3) psychological support to caregivers (session 12), and (4) care planning and coordination (session 22). Trained volunteers, supervised by the trained care managers, form the second tier of intervention. They mainly provided in-home services, including (1) psychoeducation (sessions 13, 14, 15, 16, and 17), (2) skill-building (sessions 8, 9, 10, and 11), and (3) social support (sessions 18, 19, 20, and 21). Stroke survivors also took part in up to 4 sessions (sessions 18, 19, 20, and 21) if they agreed and were competent to take part in the intervention. However, the participation of the stroke survivors did not affect their caregivers’ involvement in the study and the intervention. Although the intervention was individualized and tailor-made, the research team provided a standardized protocol to guide the trained care managers as well as the trained volunteers in delivering the intervention. Further, the interventionists recorded the sessions conducted as well as the duration of each session in a service log. Because the collaborating NGOs provided social services or rehabilitation services to stroke survivors, rapport building between care managers and stroke families was ensured. All participating stroke families agreed on the intervention and signed consent forms. Further, care managers built a constant feedback mechanism with the participants, which can ensure participant adherence.

**Table 2:** Domains and themes of the intervention sessions.

Domain	Theme	Interventionist	
		Care Manager	Volunteer
Family Needs Assessment	Project orientation, family needs assessment, and goal setting	✔	
Family Support I	Family conference	✔	
Family Support II	Addressing relationship and caregiving issues of spousal caregiver	✔	
Family Support III	Addressing relationship and caregiving issues of adult-child caregiver	✔	
Family Support IV	Rediscovery of family strengths	✔	
Family Support V	Emotion management	✔	
Family Support VI	Empower stroke survivor	✔	
Skill Building I	Caregiver self-care and relaxation		✔
Skill Building II	Mastering care skills		✔
Skill Building III	Communicating with stroke survivor with communication or swallowing problems		✔
Skill Building IV	Communicating with health care professionals		✔
Skill Building V	Stress coping and problem-solving	✔	
Psychoeducation I	Understand poststroke health issues		✔
Psychoeducation II	Medication management		✔
Psychoeducation III	Home safety and emergency handling		✔
Psychoeducation IV	Promoting healthy lifestyle		✔
Psychoeducation V	Navigating community resources		✔
Social Support I	Speech training		✔
Social Support II	Cognitive stimulating activities		✔
Social Support III	In-home rehabilitation exercise		✔
Social Support IV	Family outing	✔	✔
Social Support V	Care plan and coordination	✔	

#### Volunteer-Led Psychoeducation (Control Group)

The control group received a standard, non–family-based psychoeducation intervention provided by trained volunteers under the supervision of trained care managers. The intervention lasted for 2 months with 4 weekly sessions held at the homes of the caregivers of stroke survivors in the first month and 2 telephone contacts made in the second month (6 contact points in total). Each session lasted for 60-90 minutes. Care managers did not provide any direct intervention for participants in the control group. Stroke survivors were not involved in the control group intervention. Intervention in the control group consisted of 4 weekly home visits by trained volunteers who provided psychoeducation to caregivers, including (1) medication management and communication with health care professionals, (2) healthy lifestyle, (3) stroke care skills, and (4) stroke rehabilitation knowledge. Finally, 2 telephone contacts were made by trained volunteers to educate caregivers on (1) navigating community resources, and (2) preventing recurrent stroke.

### Outcomes

#### Primary Outcome Measures

The primary outcome revolves around the family level. The family-level outcome measures to be obtained include the Family Role Performance Scale, Care Management Strategies Scale, FCCS, and Family Assessment Device-General Functioning (FAD-GF) scale. The details of each scale are described below (Table 3).

**Table 3:** Outcome measures.

Outcome measures			Baseline		First follow-up		Second follow-up		Six months after second follow-up
**Primary outcomes**									
	Family Role Performance Scale	✔		✔		✔			
	Family Assessment Device-General Functioning Scale	✔		✔		✔			
**Secondary outcomes**									
	Care Management Strategies Scale	✔		✔		✔			
	Family Caregiver Conflict Scale	✔		✔		✔			
	Cantonese short version of Zarit Burden Interview	✔		✔		✔			
	The Patient Health Questionnaire-9 item	✔		✔		✔			
	Caregiving Ambivalence Scale	✔		✔		✔			
	Social care services used by stroke survivors	✔		✔		✔			
	Medical care services used by stroke survivors	✔		✔		✔			
	Clinical profile and service use data of stroke survivors							✔	
	Cost of study participation							✔	

#### Family Role Performance Scale

The research team developed this scale to measure the frequency and ability of the family member to perform 6 major family roles, that is, advisor, emotional connector, breadwinner, caretaker, decision maker, and caregiver by using the structure developed and validated by Chen et al [19]. This scale contains 2 parts. The first part contains 6 items and asks participants how often they perform the 6 family roles. Each item is answered on a scale ranging from 0 (*never*) to 4 (*very frequently*). The second part contains 6 items and asks participants to rate their performance regarding family roles. Each item is answered on a scale ranging from 0 (*very poor*) to 4 (*very good*). The 2 parts will be scored separately.

#### Care Management Strategies Scale

The research team developed this scale to measure caregivers’ care management strategies. The Care Management Strategies Scale consists of 18 items describing care management behaviors, including both positive and negative aspects, developed by the research team. The positive part contains 9 items and asks participants how often they perform positive care management strategies. Each item is answered on a scale ranging from 0 (*never*) to 4 (*very frequently*). The negative part contains 9 items and asks participants how often they perform negative care management strategies. Each item is answered on a scale ranging from 0 (*never*) to 4 (*very frequently*). The 2 parts will be scored separately, and then reverse coding will be applied to the negative part to provide a composite score. A higher score indicates better management strategies.

#### FCCS

The FCCS consists of 15 items to assess family conflict due to stroke. Each item is answered on a scale ranging from 1 (*strongly disagree*) to 5 (*strongly agree*). It has 4 subscales, namely, communication, problem-solving, general family functioning, and perceived criticism [16].

#### FAD-GF Scale

The FAD-GF scale is the 12-item general functioning of the McMaster Family Assessment Device [21] to measure the family functioning of caregivers. Each item is answered on a scale ranging from 1 (*strongly agree*) to 4 (*strongly disagree*).

#### Secondary Outcome Measures

The secondary outcome is related mainly to the personal level. Personal-level outcome measures include the Cantonese Short Version of Zarit Burden Interview (CZBI-short), Patient Health Questionnaire-9 item (PHQ-9), and Caregiving Ambivalence Scale. The study team will also obtain service utilization records and perform a cost-effectiveness evaluation. The details of each secondary outcome measure are described above (Table 3).

#### CZBI-Short

The CZBI-short is a spoken Cantonese version of the 12-item ZBI to assess the burden on Chinese dementia caregivers in clinical and social care settings [22]. Each item is answered on a scale ranging from 0 (*never*) to 4 (*very frequently*).

#### PHQ-9

The PHQ-9 is a reliable and valid instrument for assessing depressive symptoms in the general Hong Kong population. It consists of 9 items and was developed to correspond to the Diagnostic and Statistical Manual of Mental Disorders (Fourth Edition) criteria for major depression [23]. Each item is answered on a scale ranging from 0 (*not at all*) to 4 (*nearly every day*).

#### Caregiving Ambivalence Scale

The Caregiving Ambivalence Scale is adapted from the Intergenerational Ambivalence Scale [24] to measure the level of ambivalence between caregivers and care recipients. The scale for caregivers consists of 6 items—3 asking the positive components and 3 asking the negative components of their relationships. Each item is answered on a scale ranging from 0 (*never*) to 4 (*very frequently*).

#### Service Utilization Record

The service utilization of medical and social care services by stroke survivors was obtained. Medical care services include inpatient hospital admission, specialist outpatient, accident and emergency service, and hospital rehabilitation service. Social care services include home care service, daycare service, community rehabilitation service, and residential care service (admission after study intake). The clinical profile and service use data of stroke survivors and the electronic medical records from the Hospital Authority Clinical Management System of Hong Kong will be retrieved regularly until 6 months after the completion of the second follow-up assessment.

#### Cost and Cost-effectiveness Evaluation

In this study, the cost of study participation, including staff cost, travel expenses, and program materials, will be calculated. The research team will use the incremental cost-effectiveness ratio as well as the additional cost incurred to bring about one additional unit of a positive outcome to evaluate cost-effectiveness.

### Statistical Analysis

We will use descriptive statistics to present baseline characteristics and outcome measures, and we will transform any skewed variables and correct their skewness before inferential analysis. To assess normality, we will perform the Shapiro-Wilk test of normality as well as the normal Q-Q Plot, while we will use the Wilcoxon signed-rank test for the nonparametric test. We will also perform the chi-square or independent two-tailed *t* tests to examine the differences in the baseline characteristics between the intervention and control groups. For the primary and secondary outcomes, we will perform a multi-factor analysis of variance or general linear model to compare the differential changes in each outcome across the 3 assessment time points, that is, T0, T1, and T2 between the 2 treatment groups. This model can account for repeated measures data that are intercorrelated and that produce unbiased estimates even in the presence of the missing data, provided that the data are missing at random. Multivariate regression will be used to compare the differences in the outcomes between the 2 treatment groups and will control for the effect of potential covariates such as depressive symptoms and caregiver burden. Recruitment rate, attrition rate, and missing data will also be examined and reported. All outcome measures will be analyzed based on intention-to-treat principles. Every participant who is randomized according to the randomized treatment assignment will be included. This method of analysis preserves the prognostic balance afforded by randomization [25]. The cost of study participation, including staff cost, travel expenses, and program materials, will be expressed in Hong Kong dollars. All the statistical analyses will be performed using SPSS Statistics. All statistical tests will be two-sided with the level of significance set at .05.

### Data Handling and Record Keeping

Hardcopies of the data forms will be anonymized after data entry and will be stored in a locked cabinet inside the premises at The University of Hong Kong (2/F, HKJC Building for Interdisciplinary Research). Electronic personal data will be stored in a secured server of the university. The principal investigator will be responsible for safekeeping of personal data during and after the study. The data will be used for academic and clinical research only and will be kept for up to 5 years after the first publication. This study will inform the participants that the institutional review board and ethics committee authority will have access to source data or documents related to this study directly to monitor and review the study.

## Results

The clinical trial began recruiting in January 2017, and recruitment was completed at the end of April 2019. Data collection and data set construction were completed at the end of March 2020. As of March 2020, enrollment has been completed (n=264 stroke caregivers). A total of 200 participants completed the baseline questionnaires. We aim to publish the results by mid-2021. The intervention protocol is in Traditional Chinese only and is available upon request from the corresponding author.

## Discussion

This is the first randomized controlled trial to investigate the effectiveness and cost-effectiveness of a two-tier family-oriented intervention that utilized trained care managers and trained volunteers to support stroke caregivers in Hong Kong. The study results will provide evidence on the feasibility, sustainability, and monetary value, and thus inform stakeholders. Stakeholders include policymakers in various government bureaus and departments such as Labor and Welfare Bureau, Social Welfare Department, and Hospital Authority; health care professionals; social work professionals, including those who provide services in the government, NGOs, as well as the private sector; semiformal caregivers such as foreign domestic helpers; stroke caregivers, stroke patients, and stroke families; and media and social media, on decisions in integrating this new model into the current stroke care services. This intervention attempts to fill a service gap in the current stroke care system and serves as an important basis on which future evidence-based programs supporting family caregivers of stroke survivors could be developed.

This study is not without limitations. First, the latest official statistics that we could retrieve was those of 2016 when this study began. Subsequent statistical data related to stroke patients and caregivers were not available; therefore, statistical data obtained in 2016 formed the basis of our study design. Another limitation is the potential bias and the effect of confounding, but we provided standardized training to all care managers and volunteers to minimize intervention bias.

## Appendix

Multimedia Appendix 1 Demographics of the stroke caregivers and patients with stroke in the randomized controlled trial with 2 groups.

## Abbreviations

CZBI-short: Cantonese short version of Zarit Burden Interview
FAD-GF: Family Assessment Device-General Functioning
FCCS: Family Caregiver Conflict Scale
NGO: nongovernmental organization
PHQ-9: Patient Health Questionnaire-9 item
