# Supplementary material for: Effectiveness of a Two-Tier Family-Oriented Intervention in Enhancing the Family Functioning and Care Capacity of the Family Caregivers of Stroke Survivors: Protocol for a Randomized Controlled Trial
Source: JMIR Res Protoc. 2021 May 28;10(5):e16703. doi: 10.2196/16703 (PMC8196356; doi:10.2196/16703)
Supplement: Multimedia Appendix 1 [file resprot_v10i5e16703_app1.docx]

Demographic data comparison between caregivers in care management and volunteer-only group

|  | Care management group | | | Volunteer-only group | | | Statistics | *p* |
| --- | --- | --- | --- | --- | --- | --- | --- | --- |
|  | n | frequency | % | n | frequency | % |  |  |
| **Caregivers** |  |  |  |  |  |  |  |  |
| Gender | 100 |  |  | 100 |  |  |  |  |
| Male |  | 24 | 24.0 |  | 34 | 34.0 | X^2^(1) = 2.43 | .119 |
| Female |  | 76 | 76.0 |  | 66 | 66.0 |  |  |
| Age group | 100 |  |  | 100 |  |  |  |  |
| Under 49 |  | 31 | 31.0 |  | 27 | 27.0 | X^2^(3) = .45 | .930 |
| 50-59 |  | 30 | 30.0 |  | 33 | 33.0 |  |  |
| 60-69 |  | 24 | 24.0 |  | 24 | 24.0 |  |  |
| 70 or above |  | 15 | 15.0 |  | 16 | 16.0 |  |  |
| Marital status | 100 |  |  | 100 |  |  | X^2^(4) = 1.37 | .850 |
| Single |  | 18 | 18.0 |  | 17 | 17.0 |  |  |
| Married |  | 79 | 79.0 |  | 80 | 80.0 |  |  |
| Partner |  | 1 | 1.0 |  | 0 | 0.0 |  |  |
| Widowed |  | 1 | 1.0 |  | 1 | 1.0 |  |  |
| Divorced |  | 1 | 1.0 |  | 2 | 2.0 |  |  |
| Education level | 100 |  |  | 100 |  |  | X^2^(5) = 3.54 | .617 |
| No formal education |  | 7 | 7.0 |  | 12 | 12.0 |  |  |
| Primary |  | 25 | 25.0 |  | 22 | 22.0 |  |  |
| Secondary |  | 46 | 46.0 |  | 43 | 43.0 |  |  |
| Post-secondary |  | 7 | 7.0 |  | 12 | 12.0 |  |  |
| Bachelor |  | 11 | 11.0 |  | 8 | 8.0 |  |  |
| Master or above |  | 4 | 4.0 |  | 3 | 3.0 |  |  |
| Employed |  | 39 | 39.0 |  | 37 | 37.0 | X^2^(1) = .09 | .771 |
| Source of income |  |  |  |  |  |  |  |  |
| CSSA |  | 9 | 9.0 |  | 9 | 9.0 | X^2^(1) = .00 | 1.000 |
| NDA |  | 1 | 1.0 |  | 4 | 4.0 | X^2^(1) = 1.85 | .174 |
| HDA |  | 1 | 1.0 |  | 3 | 3.0 | X^2^(1) = 1.02 | .312 |
| OAA |  | 2 | 2.0 |  | 3 | 3.0 | X^2^(1) = .21 | .651 |
| OALA |  | 9 | 9.0 |  | 9 | 9.0 | X^2^(1) = .00 | 1.000 |
| Insurance |  | 1 | 1.0 |  | 0 | 0.0 | X^2^(1) = 1.01 | .316 |
| Pension |  | 9 | 9.0 |  | 6 | 6.0 | X^2^(1) = .65 | .421 |
| Family support |  | 26 | 26.0 |  | 31 | 31.0 | X^2^(1) = .61 | .434 |
| Salary^#^ |  | 38 | 38.0 |  | 39 | 39.0 | X^2^(1) = .02 | .884 |
| Savings |  | 23 | 23.0 |  | 28 | 28.0 | X^2^(1) = .66 | .417 |
| Others |  | 2 | 2.0 |  | 1 | 1.0 | X^2^(1) = .34 | .561 |
| Monthly income | 99 |  |  | 97 |  |  | X^2^(1) = .06 | .802 |
| Below HK$10000 |  | 68 | 68.7 |  | 65 | 67.0 |  |  |
| Above HK$10000 |  | 31 | 31.3 |  | 32 | 33.0 |  |  |
| Housing situation | 100 |  |  | 100 |  |  | X^2^(5) = 6.91 | .228 |
| Public |  | 33 | 33.0 |  | 39 | 39.0 |  |  |
| Subsidized |  | 19 | 19.0 |  | 23 | 23.0 |  |  |
| Private |  | 32 | 32.0 |  | 28 | 28.0 |  |  |
| Rental (entire) |  | 5 | 5.0 |  | 6 | 6.0 |  |  |
| Rental (partial) |  | 1 | 1.0 |  | 2 | 2.0 |  |  |
| Others |  | 10 | 10.0 |  | 2 | 2.0 |  |  |
| Living arrangement |  |  |  |  |  |  |  |  |
| Alone |  | 2 | 2.0 |  | 1 | 1.0 | X^2^(1) = .34 | .561 |
| Child / -in-law |  | 40 | 40.0 |  | 42 | 42.0 | X^2^(1) = .08 | .774 |
| Grandchild |  | 4 | 4.0 |  | 3 | 3.0 | X^2^(1) = .15 | .700 |
| Spouse / Partner |  | 76 | 76.0 |  | 72 | 72.0 | X^2^(1) = .42 | .519 |
| Parents |  | 21 | 21.0 |  | 22 | 22.0 | X^2^(1) = .03 | .863 |
| Siblings |  | 12 | 12.0 |  | 9 | 9.0 | X^2^(1) = .48 | .489 |
| Domestic helpers |  | 18 | 18.0 |  | 11 | 11.0 | X^2^(1) = 1.98 | .160 |
| Others |  | 4 | 4.0 |  | 2 | 2.0 | X^2^(1) = .69 | .407 |
| Attended formal course |  | 12 | 12.0 |  | 13 | 13.0 | X^2^(1) = .05 | .831 |
| First time caregiving |  | 90 | 90.0 |  | 88 | 88.0 | X^2^(1) = .20 | .651 |
| Joined support group |  | 2 | 2.0 |  | 3 | 3.0 | X^2^(1) = .19 | .667 |
|  | n | Mean | SD | n | Mean | SD | Statistics | *p* |
| Duration of caregiving | 93 | 30.53 | 83.43 | 100 | 12.71 | 27.59 | t(191) = 1.96 | .051 |

n=number of cases with data available. SD= Standard deviation.

Demographic and T1 data comparison between patients in care management and volunteer-only group

|  | Care management group | | | Volunteer-only group | | | Statistics | *p* |
| --- | --- | --- | --- | --- | --- | --- | --- | --- |
|  | n | frequency | % | n | frequency | % |  |  |
| **Patients** |  |  |  |  |  |  |  |  |
| Gender | 100 |  |  | 100 |  |  |  |  |
| Male |  | 56 | 56.0 |  | 47 | 47.0 | X^2^(1) = 1.62 | .203 |
| Female |  | 44 | 44.0 |  | 53 | 53.0 |  |  |
| Education level | 99 |  |  | 100 |  |  |  |  |
| No formal education |  | 21 | 21.2 |  | 30 | 30.0 | X^2^(5) = 4.72 | .451 |
| Primary |  | 33 | 33.3 |  | 22 | 22.0 |  |  |
| Secondary |  | 37 | 37.4 |  | 41 | 41.0 |  |  |
| Post-secondary |  | 6 | 6.1 |  | 4 | 4.0 |  |  |
| Bachelor |  | 1 | 1.0 |  | 2 | 2.0 |  |  |
| Master or above |  | 1 | 1.0 |  | 1 | 1.0 |  |  |
| Employed prior to stroke |  | 37 | 37.4 |  | 32 | 32.0 | X^2^(1) = .63 | .426 |
|  | n | Mean | SD | n | Mean | SD | Statistics | *p* |
| Age | 100 | 67.68 | 13.00 | 100 | 68.23 | 15.45 | t(198) = .27 | .786 |
| Comorbidities | 96 | 2.33 | 1.50 | 94 | 2.23 | 1.51 | t(188) = -.45 | .650 |
| Medical expenses | 94 | 2729 | 6404 | 90 | 2433 | 4351 | t(182) = -.37 | .715 |
| Transport expenses | 93 | 306 | 589 | 85 | 366 | 1056 | t(176) = .47 | .638 |
| Escort service expenses | 90 | 80 | 737 | 82 | 191 | 1677 | t(170) = .57 | .568 |
| smRSq total | 100 | 3.15 | 1.47 | 100 | 3.26 | 1.46 | t(198) = .53 | .596 |
| MRSv2 total | 85 | .58 | .76 | 82 | .73 | .85 | t(165) = 1.25 | .214 |
| ADL total | 89 | 66.65 | 29.46 | 90 | 60.64 | 28.41 | t(177) = -1.39 | .167 |
| IADL total | 98 | 5.64 | 5.16 | 99 | 4.46 | 4.03 | t(195) = -1.79 | .076 |
| Perceived health | 81 | 1.75 | 1.03 | 86 | 1.50 | .98 | t(165) = -1.63 | .106 |
| CRPHQ9 total | 79 | 8.28 | 6.13 | 83 | 7.35 | 5.52 | t(160) = -1.02 | .312 |
| MOCA total | 69 | 19.23 | 6.89 | 64 | 20.11 | 7.72 | t(131) = .72 | .473 |

n=number of cases with data available. SD= Standard deviation.
